# From silence to sound: A journey through hearing loss, advocacy and academia

**DOI:** 10.4102/ajod.v15i0.1822

**Published:** 2026-02-27

**Authors:** Hanri Kriel, Maria M. Kriel-Kruger

**Affiliations:** 1 Department of Drama, School of the Arts, Faculty of Humanities, University of Pretoria, Pretoria, South Africa; 2 Independent Researcher, Pretoria, South Africa

## Introduction

This article reflects on my life as a woman with a hearing impairment who lives in a predominantly auditory world. My journey with hearing loss, stemming from Miller syndrome – a rare condition affecting hearing, facial features and limb abnormalities (Van Roey et al. 2025) – has significantly influenced my life, shaping both my academic and professional pursuits. By sharing my personal story and my mission to achieve my PhD dream, I aim to highlight the challenges I have faced as an individual who is hard-of-hearing, particularly while working within the South African television industry. This article intertwines personal experiences with academic insights to advocate for greater inclusivity and accessibility in media.

## A mother’s perspective: The early years

Hanri was born on 29 July 1991, at 19:30 in Unitas Hospital, Pretoria, via a caesarean section. At the time, we lived in Groblersdal, which only had a provincial hospital. I was working as a registered nurse in Pretoria until I went on maternity leave. During the operation, I was under anaesthesia, and when I woke up, my husband was sitting by my bedside. The first thing I asked was what she looked like. He gently told me that there were a few complications – her fingers were fused together (syndactyly), and her lower jaw appeared slightly shorter than usual. The doctors assured us these features could be corrected with surgery.

Weighing just 2.64 kg, Hanri was very small. The first 30 days of her life were spent in the neonatal Intensive Care Unit (ICU), where she faced multiple challenges, including difficulties with feeding because of her inability to coordinate sucking, swallowing, and breathing. A feeding tube was necessary, and she developed pneumonia from milk aspiration while bottle-feeding. Despite these difficulties, she showed incredible resilience.

When Hanri was just 10 days old, we visited the Oral and Maxillofacial Department at the old Hendrik Verwoerd Hospital, Pretoria, to see Professor Kurt Bütow, who became a trusted point of contact on our medical journey. We also met with Professor Gericke at the Genetics Department, where Hanri was diagnosed with Miller syndrome – a rare condition affecting facial and limb development, which occurs in approximately 1 per 1 000 000 live births (Young & Ng 2023:4). This syndrome is characterised by craniofacial and limb anomalies and conductive hearing loss because of middle ear abnormalities (Van Roey et al. 2025; Young & Ng 2023:4).

At the time, Hanri was the only person in South Africa diagnosed with this syndrome, based on the available information in a reference book, as there was no Google in 1991. Miller syndrome is caused by mutations in the Dihydroorotate Dehydrogenase (DHODH) gene, which encodes the enzyme dihydroorotate dehydrogenase, located on chromosome 16 (Kinoshita et al. 2011:506). The condition’s impact on facial development included underdeveloped cheekbones, a small lower jaw and a shortened soft palate, which contributed to Hanri’s feeding difficulties. Despite these challenges, we were determined to provide her with the best care and support, making frequent trips to Pretoria for follow-ups and therapy. Hanri’s resilience during those early months was remarkable, and as her mother, I was committed to helping her thrive in every way possible.

## Growing up with hearing loss

Growing up with a rare disability and hearing loss meant facing the everyday reality of being stared at, ridiculed by peers and struggling with communication barriers. Although these challenges were a normal part of my life, they made navigating the world particularly difficult and isolating. However, they also fuelled my determination to persevere and achieve my dreams, profoundly shaping my personal, professional and academic experiences.

From the age of 4 months, various hearing aids were suggested and fitted to address my profound hearing loss. Initially, I was fitted with a bone-anchored hearing aid (BAHA), which is placed beside the ear and transmits sound through bone conduction (Galea 2021). Because of the smaller size of my ears, finding suitable hearing aids was always challenging, requiring a tailored approach from my audiologists. As I grew older, my audiologist recommended and fitted me with six different pairs of Phonak hearing aids over the years. Each pair provided improved sound quality and features such as Bluetooth and telecoil functions, which were groundbreaking at the time. Eventually, I upgraded to Unitron hearing aids, which continued to enhance my auditory experience.

One memorable instance during a fitting with my Phonak Naida hearing aids involved hearing an unusual whooshing sound. After checking all settings and turning off various devices in the room, we discovered it was simply the air conditioner – a sound I had never heard before. This experience highlighted how different hearing aids can reveal new auditory experiences, like birds singing, alarms ringing or aeroplanes flying overhead. Despite these advancements, the challenges of being hard-of-hearing have consistently shaped both my personal and professional life.

## Education, work and professional background

During my educational journey, I attended Sonitus School for the Hard-of-Hearing, a specialised school tailored to children with hearing loss situated in Pretoria, South Africa. Later, I transitioned to a mainstream high school, Wonderboom High School in Pretoria, where I graduated with two distinctions. The move to the mainstream school was prompted by the limited subject choices available at the specialised school, and I sought broader opportunities for my future academic pursuits.

After high school, unsure of my next steps, I decided to take a gap year and pursue an opportunity to au pair. Although I met all the initial requirements and was approved in the first stage of the application process, my application was ultimately denied by the company because of my hearing loss. This setback underscored the persistent barriers faced by individuals with disabilities and hearing loss, influencing my subsequent decisions and aspirations.

Despite these setbacks, I pursued a Bachelor of Arts and Honours in Communication Studies at North-West University in Potchefstroom, moving away from home to live in a hostel, which presented its own set of challenges. After completing my studies, I struggled to find employment and faced discrimination not only because of my disability but also as a woman in a male-dominated industry. My aspiration to work on cruise ships was thwarted because of my hearing loss, despite passing all required tests. Nevertheless, I secured a position at Blixem Productions while simultaneously pursuing a Master’s degree in Communication Studies which I obtained cum laude. While working in the South African television and film industry, I became acutely aware of the lack of accessibility for viewers with hearing loss in the South African television industry. This insight motivated me to pursue a PhD focusing on captioning practices in television by focusing on one of the languages available in multicultural South Africa, namely Afrikaans, with the goal of advocating for greater inclusivity and accessibility within the media landscape. After four challenging years of hard work, I am proud to have achieved a PhD in Drama and Film Studies (Kriel 2024).

My journey, both academically and professionally, highlights the intricate connection between personal experiences and academic pursuits. The challenges faced in the industry, where my impairment was deemed a hindrance, shaped my research questions, and fuelled my commitment to improving accessibility not only for Afrikaans individuals with hearing loss but also the overall television industry.

My study investigated available captioning practices in Afrikaans, proposing a standardised solution for all viewers in all languages, and highlighting the function of captions in society. Captions are the on-screen display of text that was said during the on-screen dialogue and showcasing the sound effects (Zárate 2021). Globally, captions are widely implemented, particularly for widely spoken languages. In South Africa, however, caption availability is limited, inconsistent and does not fully cover all official languages. For example, the majority of subtitles are limited to English; only a few shows make minimal use of captions, and even fewer are available in other South African languages. This highlights a significant gap in accessibility for viewers with hearing loss, underscoring the need for standardised captioning practices across all South African languages.

As a viewer with hearing loss, engaging with various soap operas and films presents unique challenges. The fast-paced dialogue, intricate plot developments, quick camera movements, off-screen narratives, reading and listening simultaneously, synchronisation, and background noise can often make it difficult to fully grasp the narrative. Watching a soap opera, a genre known for its dramatic intensity and complex character interactions, becomes a nuanced experience for those with hearing loss. Other challenges for viewers with hearing loss include subtitling inconsistencies, limited programming with subtitles and captions, the absence of standardised captioning practices as not all programmes have subtitles, and thereafter not have captions.

## Conclusion

### Towards an inclusive future – Why accessibility matters

This article emerges from a dual perspective as both a viewer and an industry professional, to emphasise the need for inclusivity in media. By sharing my journey with hearing loss, I advocate for a more accessible television industry, one that caters to the diverse needs of viewers with hearing loss. Accessibility goes beyond entertainment; it touches on fundamental rights, on allowing everyone to enjoy what many takes for granted.

Television and social media in general have become established as a significant part of life in contemporary society. The absence of readily available captions in one’s home language is a significant barrier, affecting not only content enjoyment but social inclusion for a wide variety of individuals. As technology advances, the need for accessible media becomes even more urgent to support inclusion in both online spaces and everyday media consumption.

Reflecting on my personal and professional experiences, I realise how they have influenced my academic journey. I hope that by sharing part of my story, I may, in some modest way, encourage the South African television industry to prioritise accessibility for all, thus embracing the diverse linguistic and cultural fabric of our society. By breaking the existent barriers, we can create a more inclusive media landscape that transcends language and cultural boundaries. Over time, this effort will ensure that all 12 South African languages become accessible through captions, fostering a sense of belonging and ensuring that everyone in South Africa – regardless of whether they use South African Sign Language (SASL) or spoken language – can fully engage with media content.
